# Gender Stereotype Susceptibility

**DOI:** 10.1371/journal.pone.0114802

**Published:** 2014-12-17

**Authors:** Marina A. Pavlova, Susanna Weber, Elisabeth Simoes, Alexander N. Sokolov

**Affiliations:** 1 Department of Biomedical Magnetic Resonance, Medical School, Eberhard Karls University of Tübingen, Tübingen, Germany; 2 Social and Neural System Research, Department of Economics, University of Zürich, Zürich, Switzerland; 3 Center for Women's Health, University Hospital, Eberhard Karls University of Tübingen, Tübingen, Germany; University of Bologna, Italy

## Abstract

Gender affects performance on a variety of cognitive tasks, and this impact may stem from socio-cultural factors such as gender stereotyping. Here we systematically manipulated gender stereotype messages on a social cognition task on which no initial gender gap has been documented. The outcome reveals: (i) Stereotyping affects both females and males, with a more pronounced impact on females. Yet an explicit negative message for males elicits a striking paradoxical deterioration in performance of females. (ii) Irrespective of gender and directness of message, valence of stereotype message affects performance: negative messages have stronger influence than positive ones. (iii) Directness of stereotype message differentially impacts performance of females and males: females tend to be stronger affected by implicit than explicit negative messages, whereas in males this relationship is opposite. The data are discussed in the light of neural networks underlying gender stereotyping. The findings provide novel insights into the sources of gender related fluctuations in cognition and behavior.

## Introduction

Mounting evidence points to gender impact on a variety of cognitive tasks. Overall, males robustly outperform on tasks involving spatial abilities such as mental rotation [1], [2], visual navigation [3], or parking a car: women take longer and are less accurate in forward, backward and parallel parking maneuvers [4]. Conversely, it is widely believed that females excel on social cognition and affective tasks [5]. Yet the findings are controversial: for example, contrary to common beliefs, fathers are as good as mothers at recognizing their own baby's cries [6]. Women are reported to be more sensitive to visual non-verbal cues, and exhibit superior skills in body language reading [7], but this ability is heavily modulated by emotional content of actions [8], [9]. Neuroimaging reveals the existence of gender dependent modes in the neural circuitry underpinning visual impression of social interaction [10] and body motion perception [11], [12]. Brain activation elicited by threatening facial and bodily expressions is modulated by observer's gender [13]. When healthy adults judge emotions represented by stick human body postures, patterns of recorded brain activity are sex specific [14]. This evidence supports the notion that gender differences in social cognition may have neurobiological sources [15], [16], and helps to clarify the gender specificity of a number of neurodevelopmental and psychiatric disorders [12], [17].

On the other hand, gender differences can be elicited by experience and stereotyping. From an early age, men and women are exposed to a variety of gender stereotypes, and may be treated differently or act in a different way to deal with these stereotypes [18], [19]. When studying stereotyping within the laboratory setting, participants are usually reminded that they are members of a group that is stereotypically expected to perform less well than other groups at the task in hand. In accord with this, women perform worse on a task described as a mathematical test, than on the same test described as a problem solving task [20]. Framing the task as a mathematical test likely triggers the activation of a negative stereotype in women, as women are stereotypically considered worse in mathematical tasks: performance of women is hindered when they are reminded of gender stereotypes in mathematical abilities [21], [22]. A positive (though false) stereotype message enhances performance of women on mental rotation tasks, on which females are consistently reported to be least effective [23]. When a negative stereotype is activated in a driving simulation task, women perform worse causing more accidents and “hitting” more pedestrians [24]. Performance of female soccer players decreases under the influence of the poor athletic ability stereotype [25]. Impact of stereotyping is mostly explored in women, minority groups [26] or in the elderly, in particular in respect to memory functions [27]–[29], with a minor focus on high-status, non-stigmatized groups [30]. Yet Caucasian males also under-perform when reminded of Asians' superiority in math [31], superiority of females on affective tasks [32], academic underachievement of boys [33], better verbal skills of females [34], or their low natural athletic ability [35]. Gender stereotype messages may not only enhance or diminish performance level, but can also modulate brain activity [36], and the level of sex hormones [37]. Brain mechanisms underlying stereotyping appear to be sex specific: in males, but not in females, transcranial magnetic stimulation (TMS) applied over the left dorsolateral and right anterior dorsomedial prefrontal cortices enhances gender-stereotypic bias [38].

Previous studies, however, examined gender stereotyping mostly on tasks, on which pronounced gender differences had been already established. To the best of our knowledge, only one earlier study addressed the issue of how a gender stereotype message affects performance on the task, on which no gender gap has previously been documented, and, therefore, *pure* effects of stereotyping were observed [39]. Yet messages were always formulated in explicitly positive terms (“females are usually better on the task” or “males are usually better on the task”), from which negative information might be inferred only implicitly (“if females are better on the task, then males should be worse”). Here we examined the influence of stereotype information formulated in explicitly negative terms (“females are usually worse” or “males are usually worse”) while participants performed an event arrangement (EA) task. Participants were presented with a set of cards depicting an event with human characters in a scrambled order, and had to rearrange cards into a predetermined correct sequence depicting an event in a comic-strip fashion.

## Methods

### Participants

One hundred seventeen young adults, students of the University of Tübingen (aged 20–31 years) participated in the study. They were assigned to one of three groups. One (control) group consisted of 23 participants (13 females, aged 21±0.95 years, median±95% confidence interval, and 10 males, aged 22±0.77 years; with no age differences between females and males, Mann-Whitney test, *U* = 78, n.s.). The second group included 43 participants (29 females, aged 22±1.21 years, and 14 males, aged 23.5±0.98 years; with no age differences between females and males, *U* = 225.5, n.s.). The third group involved 51 participant (25 females, aged 23±1.13 years, and 26 males, aged 23.5±1.07 years; with no age differences between females and males, *U* = 295, n.s.). They were run individually. All participants had normal or corrected-to-normal vision and heterosexual orientation. None had a history of neurological or psychiatric disorders including autistic spectrum disorders (ASD) or regular medication. None had previous experience with such tasks. The study was conducted in line with the Declaration of Helsinki and was approved by the local Ethics Committee at the University of Tübingen Medical School. Informed written consent was obtained from all participants. Participation was voluntary, and the data were processed anonymously.

### Task and procedure

The Event Arrangement (EA) test was administered to all participants. The task is included in the Wechsler Intelligenztest für Erwachsene (WIE), a test battery based on the WAIS-III (Wechsler Adult Intelligence Scale-III) by David Wechsler adapted to the German population [40]. In brief, being a part of WAIS-III, the EA task is a well-established tool for psychological assessment, psychometrically standardized, and provides normative scores obtained from a large population. For this task, several sets of cards are presented to participants. These sets portray human characters, their actions, drives, intentions, and dispositions. The sets differ in their complexity ranging in the number of cards. Each set is presented in a predetermined scrambled (false) order. The participant has to rearrange cards into a predetermined correct sequence depicting an event in a comic-strip fashion, thereby showing understanding of the event represented in the pictures. It is assumed that good performance on such tasks requires understanding the characters' mental states [41], [42], [43]. For successful performance, participants need to reflect the core of the story, which is often based on veridical perception of intentions and drives of the characters involved in this particular event. Both accuracy (correct order of cards in a sequence) and time needed for an event arrangement (as a specific time limit for each set defined in accordance to the event complexity) are taken into account when assessing performance on the task. Participants are told that each set has a specific time limit for its rearrangement. For each set, the number of errors corresponds to specific raw scores given in the WIE Manual [40]. According to the WIE Manual tables that take into account the age of participant, raw values are then transformed into the standardized normative scores ranging from 1 (floor performance) through 10 (normal or typical performance for this age) to 19 (ceiling performance). The first group of participants (controls) did not receive any prior gender related message. Instead participants got a standard gender neutral instruction how to perform the task. The second group was additionally told that males usually perform worse on this task (an explicit negative message for males, and an implicit positive message for females), and the third group was provided with prior information that females usually perform worse on this task (an explicit negative message for females, and an implicit positive message for males).

## Results

**Figure 1:**
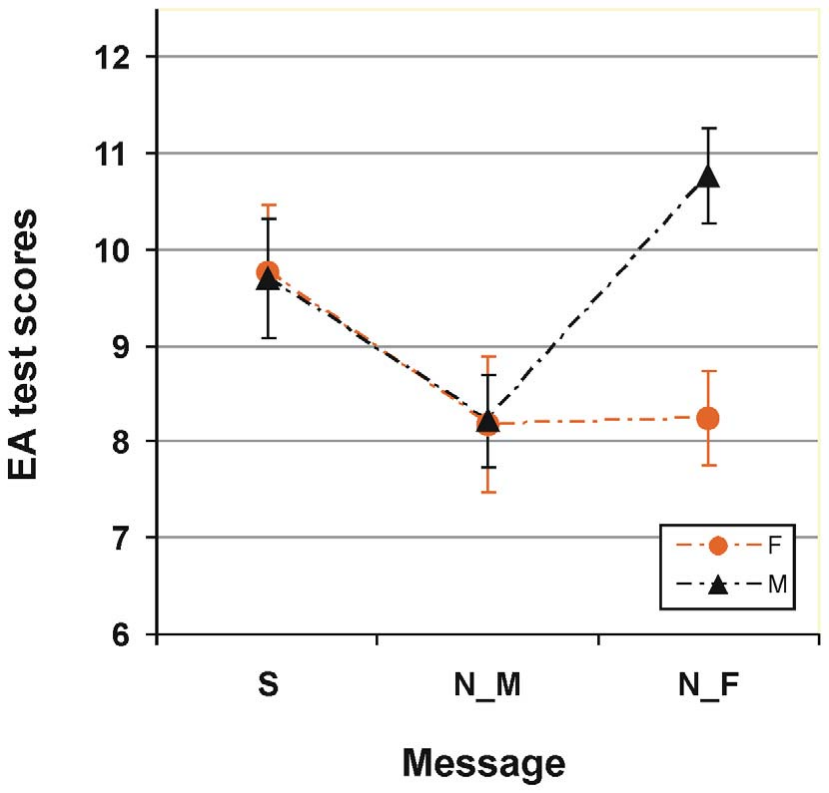
Impact of explicit negative stereotype messages on the event arrangement (EA) test. Test scores for female (represented by orange circles) and male (represented by black triangles) participants in the groups with different information given prior to testing: S, standard instruction, gender neutral message; N_F, negative for females: “Females are usually worse on this task” – an explicit negative gender stereotype message for females, and N_M, negative for males, “Males are usually worse on this task” – an explicit negative gender stereotype message for males. Vertical bars represent ±SEM.

Fig. 1 shows mean test scores on the EA task for females and males from three separate groups of participants with either standard (gender stereotype neutral), explicit negative for males or explicit negative for females instructions. The individual scores were submitted to a 2×3 two-way analysis of variance, ANOVA (as assessed by the Shapiro-Wilk test, the data were normally distributed) with between-subject factors Gender (female/male) and Message (standard/explicit negative for males/explicit negative for females). The outcome reveals a main effect of Message (*F*(2,111) = 3.74, *p*<0.027) and a Message by Gender interaction (*F*(2,111) = 3.41, *p*<0.037). As expected, without any prior gender specific information, no gender gap in performance was found (*t*(21) = 0.06, n.s.; 9.77±2.2, mean±SD, and 9.7±2.98, for females and males, respectively). As seen in Fig. 1, the explicit negative message for males (“Males are worse”) lead to a reduction in performance of males. Yet an unexpected deterioration occurs also in performance of females. This decline happens instead of an expected enhancement in performance of females as the explicit information that males are worse implicitly means that females are usually better on the task. This results in the paradoxical lack of differences in performance between male and female participants of this group (*t*(41) = 0.05, n.s.; 8.17±2.61 and 8.21±2.64, for females and males, respectively). Finally, as expected, the explicit negative information for females (“Females are worse”) leads to a deterioration in performance of females and an enhancement in performance of males resulting in a highly significant gender effect (10.78±2.52 and 8.24±2.44, for males and females, respectively; *t*(49) = 3.89, *p*<0.0003; effect size, Cohen's *d* = 1.02).

**Figure 2:**
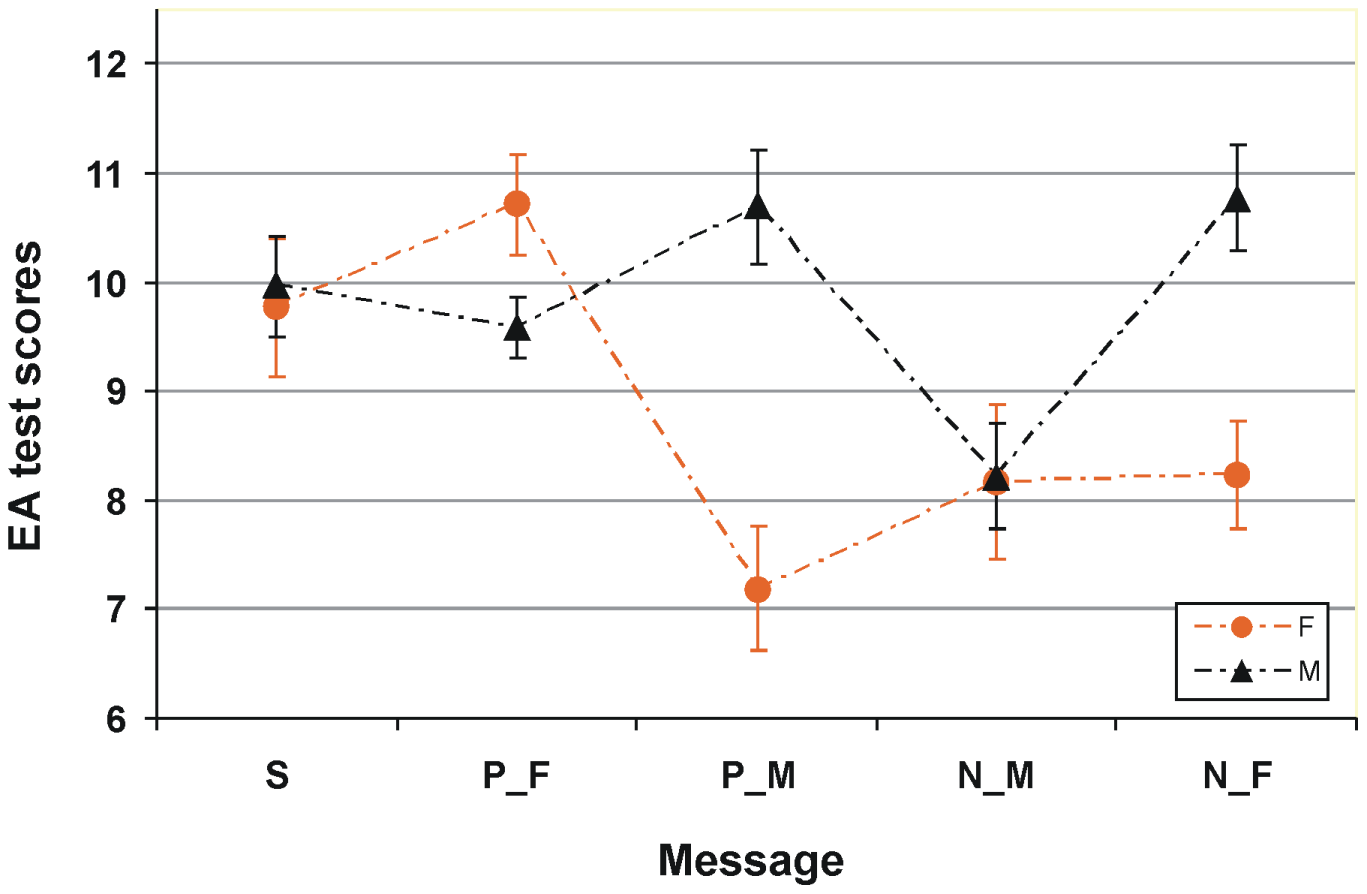
Gender stereotype susceptibility. Scores on the event arrangement (EA) test for female (represented by orange circles) and male (represented by black triangles) participants in the groups with different information given prior to testing: S, standard instruction, gender neutral message (data pooled together from control groups of the present study and [39]); P_M, positive for males, “Males are usually better on this task” – an explicit positive gender stereotype message for males (data from [39]), P_F, positive for females: “Females are usually better on this task” – an explicit positive gender stereotype message for females (data from [39]); N_M, negative for males, “Males are usually worse on this task” – an explicit negative gender stereotype message for males, and N_F, negative for females: “Females are usually worse on this task” – an explicit negative gender stereotype message for females. Vertical bars represent ±SEM.

For better understanding the impact of a gender stereotype message on fluctuations in performance, we analyzed the present data (influence of explicitly *negative* stereotype) in relation to the outcome of earlier work on explicitly *positive* stereotyping [39]. As no difference in performance occurred between females (*t*(24) = 0.00, n.s.) and males (*t*(22) = 0.34, n.s.) in the control groups of both studies, for further analysis we pooled the data for females and for males in both control groups together (Fig. 2).

The individual performance scores were submitted to a 2×5 two-way ANOVA with between-subject factors Gender (female/male) and Message (standard/positive for females/positive for males/negative for males/negative for females). The outcome reveals main effects of Message (*F*(4,192) = 3.69, *p*<0.007), Gender (*F*(1,192) = 8.21, *p*<0.005), and a Message by Gender interaction (*F*(4,192) = 5.31, *p*<0.0004).

Inspection of Fig. 2 raises the possibility that (i) overall, gender stereotype information affects females stranger than males. Furthermore, (ii) irrespective of gender and directness of message (explicit/implicit), valence (positive/negative) of stereotype information affects performance; and (iii) directness of stereotype message has a differential impact on performance of females and males: females are likely to be stronger affected by implicit than explicit negative messages, whereas males are more susceptible to explicit negative messages. We consider these assumptions in turn.

To prove if gender stereotype information affects females stronger than males, a one-way ANOVA with factor Message (standard/positive for females/positive for males/negative for males/negative for females) was conducted separately for females and males. For females, a main effect of Message was highly significant (*F*(4,105) = 6.54, *p*<0.0001). This effect was also significant for males (*F*(4,87) = 2.6, *p*<0.04). Although both females and males are affected by gender stereotype messages, females exhibit greater fluctuations in performance than do males (*p*<0.04).

For verification of assumptions (ii) and (iii), we conducted a 2×2×2 three-way ANOVA with factors Valence of message (positive/negative), Directness of message (explicit/implicit), and Gender (female/male). The outcome revealed a main effect of Valence of message (*F*(1,144) = 20.34, *p*<0.0001, with lower scores for negative information), Gender (*F*(1,144) = 9.79, *p*<0.002, with lower scores for females), and an interaction of Directness of message by Gender (*F*(1,144) = 19.21, *p*<0.002). The interaction indicates that the influence of directness is modulated by gender: Irrespective of valence of message, females are stronger affected by implicit messages (*t*(82) = 2.73, *p*<0.008; Cohen's *d* = 0.603), whereas males exhibit a trend to be stronger affected by explicit messages (*t*(66) = 1.74, *p*<0.09). Furthermore, as seen in Fig. 2, females tend to be stronger impacted by implicit rather than explicit negative stereotype messages (*t*(39) = 1.83, *p*<0.08; comparison of females in the group with an explicit negative message for females with females in the group with an explicit positive message for males, i.e. an implicit negative message for females). By contrast, males tend to be stronger affected by explicit as compared with implicit negative messages (*t*(24) = 1.95, *p*<0.06; comparison of males in the group with an explicit negative message for males with males in the group with an explicit positive message for females, i.e. implicit negative message for males). As reported earlier [39], an implicit negative message causes a dramatic decrease in performance of females as compared with males. By contrast, no difference in performance was found between females and males provided with an explicit gender-specific negative message (*t*(37) = 0.03, n.s.).

Due a paradoxical decline in performance of females under influence of an implicit positive message, females are much stronger (albeit in the opposite direction) affected by an implicit positive message than by an explicit one. As seen in Fig. 2, this results in a highly significant difference in performance of these groups (*t*(41) = 4.52, *p*<0.0001; comparison of females in the group with an explicit positive message for females with females in the group with an explicit negative message for males, i.e. with an implicit positive message for females). By contrast, in males, no difference in performance was found between the groups with explicit and implicit positive information (*t*(40) = 0.11, n.s.; comparison of males in the group with an explicit positive message with males in the group with an explicit negative message for females, i.e. with an implicit positive message for males; Fig. 2).

## Discussion

The present study explores the impact of gender-specific stereotype messages on performance. The outcome indicates that explicit and implicit gender stereotype messages affect performance of both females and males albeit in a differential way. As compared with previous work, e.g., [21]–[23], [36], here the effects of stereotype messages were found on a task on which no initial gender differences have been previously documented. Therefore, we demonstrated *pure* effects of gender stereotyping on performance. Furthermore, earlier studies did not systematically investigate and directly compare the magnitude of gender-specific stereotype effects.

Most important outcome of the study is that gender stereotype information can elicit pronounced gender differences in performance on tasks with no initial gender gap. Taken together, the findings indicate: (i) Gender related stereotype messages affect both females and males, with a more pronounced impact on females. (ii) Irrespective of gender and message directness, the valence of a gender stereotype message affects performance: negative messages stronger affect performance than do positive messages. (iii) Directness of stereotype message has differential impact on performance of females and males: females tend to be stronger affected by implicit than by explicit messages, whereas in males this relationship is opposite. Yet the most arresting finding is that an explicit negative stereotype message for males elicits paradoxical deterioration in performance of females.

The finding that females are stronger affected by implicit as compared to explicit negative messages fits well with previous reports on the stereotype threat [44]. Stereotype threat was initially defined as the fear of confirming a negative stereotype about a group to which one belongs [26]. In females, subtle threat-activating cues produce the largest effect, followed by blatant and moderately explicit cues [45]. One possible explanation for this is that implicit messages affect performance subconsciously, while explicit cues activate defense mechanisms that react against negative information. Stereotype reactance, a tendency to behave in a manner inconsistent with a stereotype, may explain cases where an explicit negative message leads to an improvement of performance [46], and may also explain why explicit cues result in a lesser effect than implicit cues. In contrast, the present data show that men are most vulnerable to explicit negative messages. This suggests different mechanisms at work in minority groups (women as traditional targets of stereotypes) and majority groups (men as traditional non-targets of stereotypes) responding to explicit and implicit stereotypes. One probable account for this is that stereotype targets have a history of stigmatization while non-targets do not have this past traumatic experience of being typecast.

First and foremost, why do negative stereotype messages appear to be more powerful than positive messages? Brain imaging indicates that stereotype messages affect neural processing, with positive messages leading to the recruitment of efficient processing strategies, and negative stereotype cues leading to less efficient ones. For example, during a challenging mathematical test, women in stereotype neutral conditions activate the brain areas known to subserve mathematical processing, whereas women under stereotype threat do not [47]. Similarly, during a mental spatial rotation task, women under control or positive stereotype conditions exhibit activation in brain areas associated with visual processing and working memory, while under stereotype threat the brain areas associated with negative emotional processing such as the amygdala are most active [36]. Furthermore, stereotype conditions have been shown to increase activity in areas associated with evaluative and decision making processing, such as the ventromedial prefrontal cortex [38], [48]. Imaging findings dovetail with lesion studies indicating that ventromedial prefrontal cortical lesions eliminate implicit gender stereotyping [49]. Overall, positive stereotype messages affect brain function by recruiting efficient task-specific neural processes, whereas negative messages elicit activity in areas involved in processing of negative emotions that in turn block task-specific networks. It is essential that informing women about the possible impact of stereotype threat on cognition and behavior can eliminate detrimental effects [50]. When informed, women no longer exhibit poorer performance on a math test, even when it is framed as such. In other words, “knowing is half the battle” [20]. Yet some data in female athletes suggest that even with explicit experimental nullification of stereotype threat, performance may not change [51]. Noteworthy, sense of humor diminishes effects of stereotype threat on women's math performance apparently because humorous females feel less anxiety and frustration while taking the test [52].

The most arresting effect is that an explicit stereotype message negative for males, elicits a paradoxical deterioration in performance of females. Actually, explicit negative stereotype message for males (“males are usually worse”) should implicitly mean that females are usually better. Yet instead of performance improvement, this message caused deterioration in performance in such a way that there was no difference in performance of females and males. One possible account is that females may have misinterpreted the message in a negative way: if the task is difficult for males, then for females it would likely be even more challenging and demanding. Negative information can disrupt performance via a number of mechanisms such as a physiological stress response that impairs prefrontal processing and is largely mediated by interaction with sex hormones; a tendency to actively monitor performance; efforts to suppress negative thoughts and emotions that can lead to reduced working memory and other cognitive capacities; and lower expectations and motivation [53], [54], [55], [56]. Stereotype susceptibility as well as consequent discouragement in the related task domain, may be responsible for under-representation of females in leading positions in the fields where stereotypes are pertinent [57].

Individuals who are highly invested in the stereotyped domain or highly identify themselves with their stigmatized groups are most vulnerable to the stereotype threat [21], [53]. In other words, women who possess high identification with their gender can be stronger affected by negative gender stereotypes. For instance, under stereotype threat conditions women with high gender-identification scores perform worse than men on a math test, whereas women with low gender-identification perform comparable to men [58]. Moreover, it appears that in order to succeed in domains where stereotypes are present, individuals may be forced to psychologically dis-identify with their gender [53]. This assumption may have far-reaching societal implications.

Female susceptibility to negative information can have important applications in the health care domain. For example, it appears likely that in female patients suffering gynecologic oncological diseases, information about disease can block mechanisms supporting recovery processes. This may happen presumably due to “diagnosis threat”, a kind of stereotype threat [59], [60], [61], [62]. Elucidating the specificity of this patient population in coping with disease related negative information would help to work out the strategies of how to inform female oncological patients, and how to support them in health-related decision making. This is of importance as health policy of many countries including Germany underpins the principle of self-determination in matters of health [63]. The present work offers new perspectives for investigation of possible influence of health-related physician-patient communication on recovery potential in oncological diseases in females.

## Conclusions

In a nutshell, the present findings indicate that gender stereotype messages affect performance of both females and males albeit in a differential way. For the first time, gender stereotype susceptibility was systematically studied on a task with no initial gender gap. Most important, gender-specific stereotype information can elicit pronounced gender differences in performance. The outcome reveals: (i) Gender stereotype messages affect both females and males, with a more pronounced impact on females. (ii) Irrespective of gender and directness of message, valence of message affects performance: negative messages stronger affect performance than do positive messages. (iii) Directness of messages differentially impacts performance of females and males: females tend to be stronger affected by implicit negative messages, whereas in males this relationship is opposite. Overall, the study provides novel insights into the possible sources of gender related fluctuations in cognition and behavior.
